# The mono(catecholamine) derivatives as iron chelators: synthesis, solution thermodynamic stability and antioxidant properties research

**DOI:** 10.1098/rsos.171492

**Published:** 2018-06-06

**Authors:** Qingchun Zhang, Bo Jin, Xiaofang Wang, Shan Lei, Zhaotao Shi, Jia Zhao, Qiangqiang Liu, Rufang Peng

**Affiliations:** 1 State Key Laboratory Cultivation Base for Nonmetal Composites and Functional Materials, Southwest University of Science and Technology, Mianyang 621010, People's Republic of China; 2 School of Materials Science and Engineering, Southwest University of Science and Technology, Mianyang 621010, People's Republic of China; 3 Research Center of Laser Fusion, China Academy of Engineering Physics, Mianyang 621010, People's Republic of China

**Keywords:** catecholamine, chelator, thermodynamic stability, antioxidant

## Abstract

There is a growing interest in the development of new iron chelators as novel promising therapeutic strategies for neurodegenerative disorders. In this article, a series of mono(catecholamine) derivatives, 2,3-bis(hydroxy)-*N*-(hydroxyacyl)benzamide, containing a pendant hydroxy, have been synthesized and fully characterized by nuclear magnetic resonance, Fourier transform infrared spectroscopy and mass spectrum. The thermodynamic stability of the chelators with Fe^III^, Mg^II^ and Zn^II^ ions was then investigated. The chelators enable formation of (3 : 1) Fe^III^ complexes with high thermodynamic stability and exhibited improved selectivity to Fe^III^ ion. Meanwhile, the results of 1,1-diphenyl-2-picryl-hydrazyl assays of mono(catecholamine) derivatives indicated that they all possess excellent antioxidant properties. These results support the hypothesis that the mono(catecholamine) derivatives be used as high-affinity chelator for iron overload situations without depleting essential metal ions, such as Mg^II^ and Zn^II^ ions.

## 1. Introduction

Iron is essential to the proper functioning of most organisms, but it is toxic when present in excess. In the presence of molecular oxygen, loosely bound iron is able to redox cycle between the two stable oxidation states Fe^II^ and Fe^III^, then catalyse the production of oxygen-derived free radicals, such as hydroxyl radical, which lead to an increase in oxidative stress markers in the substantia nigra, an increase in dopamine turnover and loss of dopamine in the striatum, *α*-synuclein pathology, and Lewy-body generation and membranal degeneration in neurons [1,2], thereby inducing neurodegenerative disorders, such as Alzheimer disease and Parkinson's disease. Therefore, we needed to develop new iron chelators as novel promising therapeutic strategies [3,4].

Over the decades, the design of hexadentate chelating agents for Fe^III^ was inspired by siderophores enterobactin, a natural microbial Fe^III^ chelating agent, which possesses favourable geometric arrangement for Fe^III^ coordination preference and highest pFe^III^ value [5,6]. Lots of hexadentate chelators were synthesized based on chelate moieties of siderophores, such as catecholamine [7–10], hydroxypyridinone [11–15], and hydroxamate [16]. Most of these hexadentate chelators have a high Fe^III^ binding ability, but almost all chelating agents with high molecular weight (greater than 500), high hydrogen bond donors (greater than 5) and high hydrogen bond acceptors (greater than 10), which lead to a poor absorption, have not attracted more attention as potential therapeutic chelators. While deferiprone is a very simple bidentate structure chelator which possesses low molecular weight (139), low hydrogen bond donors (1) and low hydrogen bond acceptors (3) it satisfies design guidelines [17] for good absorption. Hence, deferiprone has good oral activity, which has been a well-researched iron chelating agent for the treatment of iron overload [18–20]. After twenty years of clinical observations we know that deferiprone can induce agranulocytosis [21]. Therefore, we urgently needed to develop new high affinity, selective, orally and nontoxic iron chelators.

**Figure 1.**
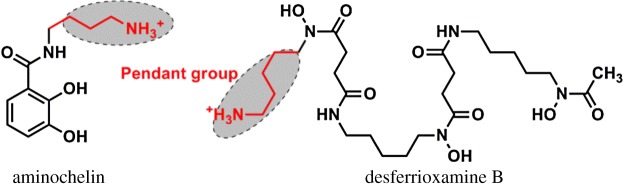
Molecular structures of desferrioxamine B and aminochelin.

Catecholamine moiety possesses a high affinity for Fe^III^. This extremely strong interaction with tripositive metal cations results from the high electron density of both oxygen atoms [22]. Therefore, an iron chelating agent which satisfies design guidelines [17] should be rationally designed, synthesized and developed based on catecholamine moiety. The new mono(catecholamine) derivatives have structural features that are unusual for a siderophore. It has a very simple bidentate structure unlike most known siderophores. Meanwhile, it has a pendant hydroxide group, which makes the ligand very hydrophilic, which is proposed to play an important role like ferrioxamine B and aminochelin (see figure 1 for structure) with a pendant amine group [23,24].

In this study, we reported the synthesis of mono(catecholamine) derivatives, the solution thermodynamic stability of these chelators with Fe^III^, Mg^II^ and Zn^II^ ions in aqueous solution and antioxidant activity.

## 2. Results and discussion

### 2.1. Synthesis and characterization

**Figure 2.**
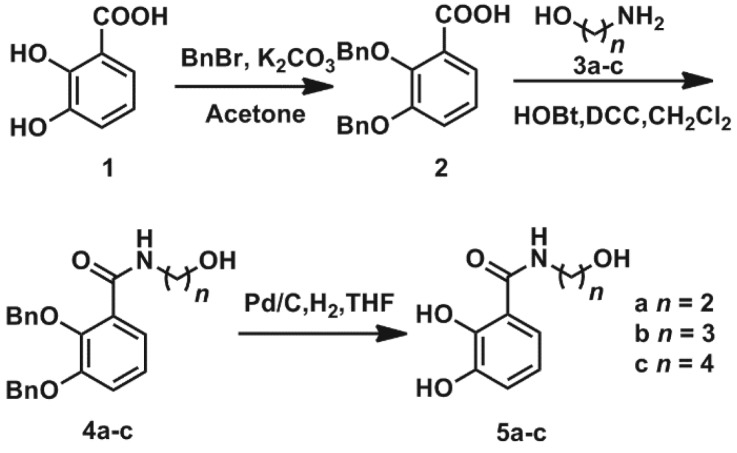
Synthesis of the mono(catecholamine) derivatives **5a–c**, L^1-3^H_2_.

The synthesis of the bidentate chelator mono(catecholamine) derivatives **4a–c** are shown in figure 2. First, 2,3-bis(dibenzyloxy)benzonic acid **2** (80%) was generated from commercially available 2,3-bis(hydroxyl)benzonic acid **1** [25]. Aminoalcohol **3a–c** and **2** were condensed using 1-hydroxybenzotriazole/dicyclohexylcarbodiimide (HOBt/DCC) to obtain the desired benzamides **4a–c** with up to 90% yield [26]. Deprotection of the hydroxyl groups under typical catalytic hydrogenation conditions with removal of the benzyl group (room temperature, 130 ml min^−1^ H_2_, atmospheric pressure and palladium on activated charcoal (Pd/C) in tetrahydrofuran) produced **5a–c** (L^1-3^H_2_) with up to 99% yield.

The mono(catecholamine) derivatives were fully characterized by nuclear magnetic resonance (MNR), Fourier transform infrared spectroscopy (FTIR) and mass spectrum (MS). The FTIR spectra of the derivatives showed distinct absorption peaks at 1639–1647 cm^−1^ of benzamide carbonyl stretching vibration, 1583–1593 and 1540–1548 cm^−1^ of benzene ring skeleton vibration. The ^1^H NMR signals of the catecholamine aromatic protons of the chelators in (CD_3_)_2_CO were identified as doublets at 7.22–7.28 ppm (d, *J* = 7.8 Hz, 1H, Ar–H), doublet of doublets at 6.96 ppm (dd, *J* = 7.8, 1.2 Hz, 1H, Ar–H) and triplets at 6.69–6.71 ppm (t, *J* = 7.8 Hz, 1H, Ar–H). The signals of methylene of benzyl at 5.15–5.16 ppm (s, 9H, O–CH_2_–Ar) and 5.09–5.10 ppm (s, 9H, O–CH_2_–Ar) disappeared. Meanwhile, the ^13^C NMR signals of methylene of benzyl also disappeared in the mono(catecholamine) derivatives. Results indicated that the structure of mono(catecholamine) derivatives is as we expected.

### 2.2. Solution thermodynamics

In its neutral form, mono(catecholamine) derivatives (hereafter also designed as L^1-3^H_2_) have two dissociable protons, corresponding to catecholamine moieties. Because catecholamine moieties require deprotonation for efficient metal chelation, their metal affinity is necessarily pH dependent. In the presence of dissolved metal ions (M^a+^) and protonated chelator (LH*_i_*, where L is a chelator with *i* removable protons), the pH-dependent metal–chelator complex with a general formula M*_m_*L*_l_*H*_h_* forms. The relative amount of each species in solution is determined by equation (2.1), whose rearrangement provides the standard formation constant notation of log *β_mlh_* (equation (2.2)). The log *β_mlh_* value describes a cumulative formation constant. For convenience, these are discussed as stepwise association constants, either for complex formation log *K_1n0_* (equation (2.3)) or ligand protonation log *K*_0*1 h*_ (equation (2.4)). When addressing protonation constants, the stepwise formation constants are commonly reported as protonation constants (log *K*_h_^H^, *h* = 1, 2, 3, … ):

2.1 $$[{\text{M}}_{m}{\text{L}}_{l}{\text{H}}_{h}]\text{=}\beta_{mlh}{\left[\text{M}\right]}^{m}{\left[\text{L}\right]}^{l}{\left[\text{H}\right]}^{h},$$

2.2 $$\log\;\beta_{mlh}=\log\;\left(\frac{\left[{\text{M}}_{m}{\text{L}}_{l}{\text{H}}_{h}\right]}{{\left[\text{M}\right]}^{m}{\left[\text{L}\right]}^{l}{\left[\text{H}\right]}^{h}}\right),$$

2.3 $$\log\;K_{01h}=\log\;\left(\frac{\left[\text{LH}h\right]}{\left[\text{L}{\text{H}}_{h-1}][\text{H}\right]}\right)=\log\;\left(\frac{\beta_{01h}}{\beta_{01(h-1)}}\right)$$

2.4 $$\;\text{and}\;\log\;K_{1n0}=\log\;\left(\frac{\left[\text{ML}n\right]}{\left[\text{M}{\text{L}}_{n-1}][\text{L}\right]}\right)=\log\;\left(\frac{\beta_{1n0}}{\beta_{1(n-1)0}}\right).$$

**Figure 3.**
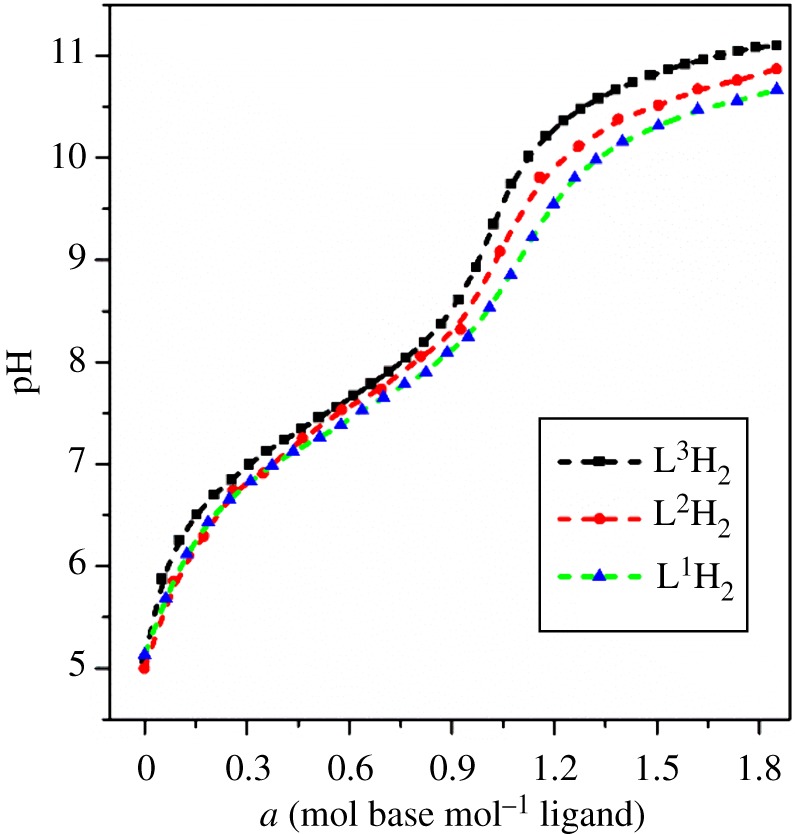
Potentiometric titration curves of L^1-3^H_2_, condition: [L^1-3^H_2_] = 5.0 × 10^−4^ M, *µ* = 0.10 M KCl, *T* = 298.2 K.

**Figure 4.**
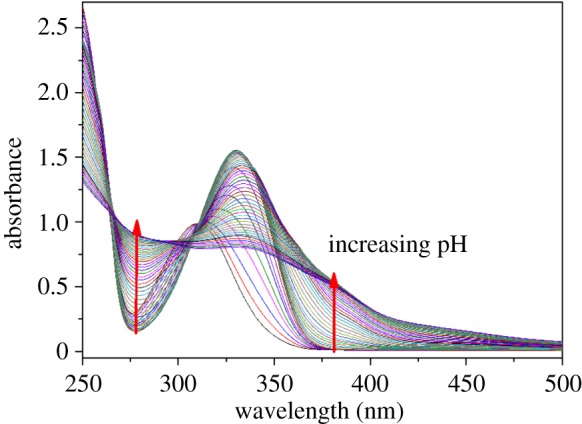
The spectrophotometric titration spectrogram of L^1^H_2_, condition: [L^1^H_2_] = 5.0 × 10^−4^ M, *µ* = 0.10 M KCl, *T* = 298.2 K, pH range = 5.3–12.5.

**Figure 5.**
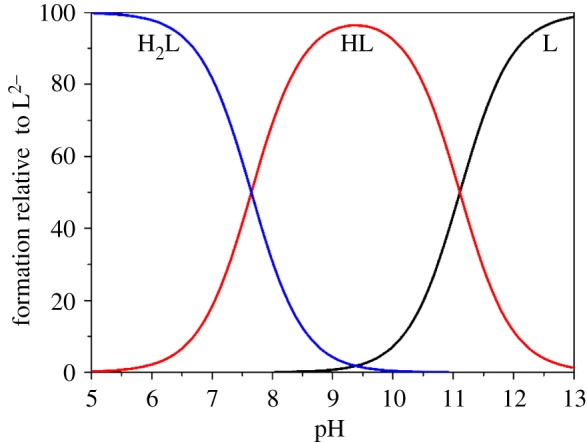
Species distribution curves calculated for the chelator L^1^H_2_, calculative condition: [L^1^H_2_] = 5.0 × 10^−4^ M, the charge numbers are omitted for clarity.

The log *K*_h_^H^ were determined from a combination of potentiometric and spectrophotometric titration. The obtained data were analysed using HypSpec 2014 and Hypquad 2013 [27,28]. The determined log *K*_h_^H^ of L^1-3^H_2_ and different phenolate type chelators are listed in table 1. All the chelators contain two basic sites from the phenolate oxygen atoms of the catechol moieties. Therefore, L^1-3^H_2_ were both treated as diprotic acids for data analysis. The potentiometric titration curves of L^1-3^H_2_ are shown in figure 3, the *a* value is the mol ratio of added base per chelators. The deprotonation of phenolic hydroxy in an orthoposition relative to amide gives rise to the buffer region at low pH (pH < 9.0), and its inflection points at *a* = 1, which indicated the orthophenolic hydroxy complete dissociation. These potentiometric titrations data were used to obtain the log *K*_2_^H^ values, whereas spectrophotometric titration data were required to accurately determine the higher log *K*_1_^H^ values. The spectrophotometric titration spectrogram of L^1^H_2_ was selected for illustration because it is similar to that of L^2-3^H_2_, as shown in figure 4. The spectrograms of L^2–3^H_2_ are shown in the electronic supplementary material, figures S1–S2. The initial absorbance peaks at 310 nm shifted to 330 nm with the increase of pH (5.3–9.0), which was attributed to the deprotonation of the phenolic hydroxy in an orthoposition relative to amide. Subsequently, the peaks width gradually broadened, and the intensity was gradually decreasing with the increase of pH (9.0–12.5), which was attributed to the further deprotonation. At pH 9.0, about 90% of L^1-3^H_2_ are in anionic form (L^1-3^H)^−^ (figure 5 and electronic supplementary material, figures S3–S4), which indicated one more lost acidic proton of catechol moiety.

**Table 1.** The log *K*_h_^H^ of L^1-3^H_2_ and different phenolate type chelators.

chelators	log *K*_1_^H^	log *K*_2_^H^
L^1^H_2_^a^	11.60	7.63
L^2^H_2_^a^	11.40	7.50
L^3^H_2_^a^	11.37	7.31
MDHB^b^	11.20	7.50
DMB^c^	12.10	8.42
catechol^d^	13.00	9.24

^a^Determined from a combination of potentiometric and spectrophotometric titrations: [L^1-3^H_2_] = 2.0 × 10^−4^ M, *µ* = 0.10 M KCl, *T* = 298.2 K.

^b^MDHB in [29].

^c^DMB in [5].

^d^Catechol in [30].

The values log *K*_2_^H^ of L^1-3^H_2_ are ascribed to the protonations of the oxygen atoms of the catecholamine dianions in an *ortho* position relative to amide. The values log *K*_2_^H^ of L^1-3^H_2_ were comparable with the corresponding values for *N*-methyl-2,3-dihydroxybenzamide (MDHB) [29], *N,N*-dimethyl-2,3-dihydroxybenzamide (DMB) [5] and catechol [30] (table 1). The results in this working were in good agreement with the value of log *K*_2_^H^=7.50 for MDHB. At the same time, the values were about one logarithm unit lower than those of DMB, which was attributed to an intramolecular hydrogen bond of the secondary amide with *ortho* position phenolate oxygen [14,31,32] moiety and the negative inductive effect of tertiary amide in catecholamine moiety. Meanwhile, the values were about two logarithm units lower than those of catechol, which not only was attributed to an intramolecular hydrogen bond [14,31,32], but also the inductive effect of the secondary amide. In fact, theoretical calculations, analysis of crystal structures and experimental potentiometric data for series of catecholamine derivatives indicated that the presence of amide on the catechol increased the second protonation constant of the nearby oxygen atoms of catechol dianions by about two logarithm units [33].

**Figure 6.**
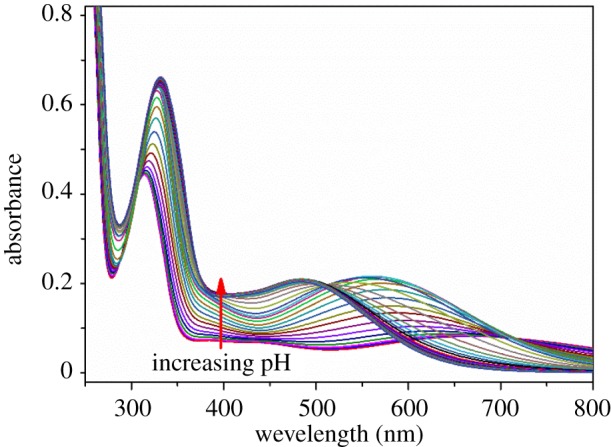
The spectrophotometric titration spectrogram of Fe^III^–L^1^H_2_ complex, condition: [L^1^H_2_] = 3 × [Fe^III^] = 2.0 × 10^−4^ M, *µ* = 0.10 M KCl, *T* = 298.2 K, pH range = 3.9–11.0.

**Figure 7.**
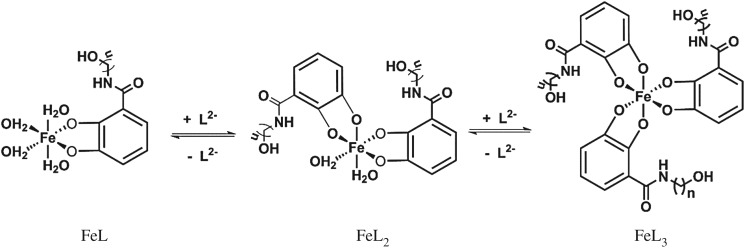
Proposed complexation processes of ligands L^1-3^H_2_, the charge numbers are omitted for clarity.

The spectrophotometric titration spectrogram of the Fe^III^–L^1^H_2_ complex was selected for illustration because it is similar to that of L^2-3^H_2_, as shown in figure 6. The spectrograms of L^2–3^H_2_ are shown in the electronic supplementary material, figures S5–S6. In the titration spectrogram, intense ligand-to-metal charge transfer (LMCT) bands of the Fe^III^ complexes allow the proton-dependent equilibria to be monitored spectrophotometrically. All of the chelator L^1-3^H_2_ possess at least three spectrophotometrically distinguishable species that can be assigned by comparison to the well-defined Fe^III^–catechol system [34–36]. For the sake of clarity, we will use the FeL*_n_* notation to ignore the charge state of the complexes, which varies across our series of chelator. In all cases, a light green solution was observed under acidic conditions, corresponding to FeL forms. Upon addition of alkali, a blue solution was observed, which is indicative of FeL_2_ predominates. The solution converts, under more alkaline conditions, to red that is assigned as FeL_3_. So, we proposed the complexation processes are that complete deprotonation bidentate gradually replaced solvent molecules with the increased pH value (figure 7). The specific species and the LMCT band wavelengths with their corresponding extinction coefficients for each FeL*_n_* spectral are compiled in table 2.

**Table 2.** Wavelength and extinction coefficients for FeL*_n_* species extracted from spectrophotometric titrations*^a^*.

	FeL		FeL_2_		FeL_3_	
chelator	*λ*_max_ (*ε*)^b^	pH	*λ*_max_ (*ε*)^b^	pH	*λ*_max_ (*ε*)^b^	pH
L^1^H_2_	660 (415)	3.9–4.8	561 (1085)	4.8–7.6	485 (1050)	>7.6
L^2^H_2_	657 (506)	3.9–4.8	558 (1220)	4.8–7.5	485 (1330)	>7.5
L^3^H_2_	655 (442)	3.9–4.7	558 (1260)	4.7–7.5	485 (1335)	>7.5

^a^The pH range indicates the calculated pH values in which that species predominates, conditions: [L^1-3^H_2_] = 3 × [Fe^3+^] = 2.0 × 10^−4^ M, *µ* = 0.10 M KCl, *T* = 298.2 K.

^b^*λ*_max_ in nm and *ε* in M^−1^ cm^−1^.

**Figure 8.**
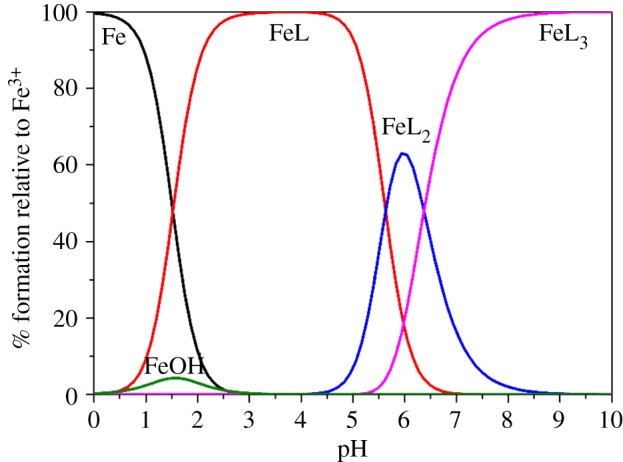
Species distribution curves of Fe^III^–L^1^H_2_ complex, calculative condition: [L^1^H_2_] = 3 × [Fe^III^] = 2.0 × 10^−4^ M, the charge numbers are omitted for clarity.

The affinities of chelator L^1-3^H_2_ with metal ions were determined by spectrophotometric titration data, which were analysed using the program HypSpec 2014 [27]. The species distribution diagrams of metal-complexes were obtained using the simulation program HySS [37]. The species distribution diagrams of Fe^III^ complexes are shown in figure 8 and the electronic supplementary material, figures S7–S8. The log *β_mlh_* of L^1-3^H_2_ with Fe^III^ are listed in table 3. But, log *β_mlh_* values are species dependent, therefore, a species-independent metric is needed to compare metal affinities of various ligands. In this regard, pM is the metric employed, where pM = −log[M_free_]. ‘M_free_’ refers to solvated metal ions free of complexation by ligands or hydroxides; high pM corresponds to low concentrations of uncomplexed metal ions in the solution. In this study, pM values are calculated using standard conditions of [M] = 10^−6^ M, [L] = 10^−5^ M and pH = 7.4. The pFe^III^ values of chelator L^1-3^H_2_ and related compounds are listed in table 3. The pFe^III^ values of the chelator L^1-3^H_2_ are significantly higher than those of deferiprone [38] and aminochelin [24]. Meanwhile, changes in minor log *K*_i_^H^ values are an insignificant factor in determining Fe^III^ affinity in chelators L^1-3^H_2_, the higher affinity of L^1^H_2_ is presumably owing to favourable geometric arrangement between the ligand and the Fe^III^ coordination preference. The fact that the pFe^III^ value of L^1^H_2_ is higher than that of L^2-3^H_2_ indicated a shorter pendant hydroxide group with lesser steric hindrance, which favours higher Fe^III^ affinity.

**Table 3.** The log *β_mlh_* and pFe^III^ of L^1-3^H_2_ and related compounds.

chelator	log *ß*_110_	log *ß*_120_	log *ß*_130_	pFe^III^^a^
L^1^H_2_	19.96(2)	31.94(3)	42.66(3)	19.37(4)
L^2^H_2_	19.23(3)	31.36(4)	41.66(2)	19.23(2)
L^3^H_2_	19.19(2)	31.04(5)	41.10(3)	19.06(4)
deferiprone^b^	15.21	26.97	36.75	20.5
aminochelin^c^	19.10	30.80	41.30	17.6

^a^pFe^III^ = −log[Fe^III^_free_], [Fe^III^] = 10^−6^ M and [L] = 10^−5^ M.

^b^Deferiprone in [39].

^c^Aminochelin in [24].

Metal affinity studies have focused on the Fe^III^ ion; however, the presence of Mg^II^ and Zn^II^ ions in biological systems leads us to evaluate the affinity of the chelators with Mg^II^ and Zn^II^ ions. The Mg^II^ and Zn^II^ ions affinities of L^1-3^H_2_ were determined through spectrophotometric titrations under the same conditions above. The spectrophotometric titration spectrograms of Mg^II^–L^1-3^H_2_ and Zn^II^–L^1-3^H_2_ complexes are shown in the electronic supplementary material, figures S9–S14. The log *β_mlh_*, pMg^II^ and pZn^II^ values of L^1-3^H_2_ and related compounds are listed in table 4.

**Table 4.** The log *β_mlh_*, pMg^II^ and pZn^II^ of L^1-3^H_2_ and related compounds.

	Mg*_m_*L*_l_*H*_h_*		Zn*_m_*L*_l_*H*_h_*	
chelator	log *ß*_110_	pMg^II^^a^	log *ß*_110_	pZn^II^^b^
L^1^H_2_	6.24(5)	6.00	10.05(2)	6.54(2)
L^2^H_2_	6.26(2)	6.00	10.08(4)	6.56(3)
L^3^H_2_	6.29(3)	6.00	10.11(1)	6.58(1)
deferiprone^c^	—	—	7.19	6.28
DTPA^d^	—	6.40	—	15.10

^a^pMg^II^ = −log[Mg^II^_free_], [Mg^II^] = 10^−6^ M and [L] = 10^−5^ M.

^b^pZn^II^ = −log[Zn^II^_free_], [Zn^II^] = 10^−6^ M and [L] = 10^−5^ M.

^c^The pZn^II^ was calculated by the log *K_i_*^H^ and log *β_mlh_* values of deferiprone in [39].

^d^DTPA in [40].

The pMg^II^ and pZn^II^ values of L^1-3^H_2_ were significantly lower than those of the efficient chelator diethylenetriaminepentaacetic acid (DTPA) [40]. The low pMg^II^ and pZn^II^ values were similar to those of catechol chelators [8,10,40–44], indicating the formation of chelators L^1-3^H_2_ with weak Mg^II^ and Zn^II^ affinity, as predicted.

### 2.3. Antioxidant activity

1,1-Diphenyl-2-picryl-hydrazyl (DPPH) is a stable free radical [45], which is widely used to monitor the free radical scavenging ability of various antioxidants [46–48]. The assays were carried out in methanol, and the results were expressed as EC_50_, which represented the antioxidant concentration required to decrease the initial DPPH concentration by 50%. Low EC_50_ values indicate a highly radical scavenging capacity. This parameter is widely used to measure antioxidant capacity but does not consider the reaction time. The time needed to reach the steady state to the concentration corresponding at EC_50_ (*T*_EC50_) was calculated, and antiradical efficiency (AE) was introduced as a parameter to characterize antioxidant compounds [46]. AE was determined by the following equation:

2.5 $$\text{AE = }\frac{1}{\text{E}{\text{C}}_{50}\times T_{E{\text{C}}_{50}}}.$$

**Figure 9.**
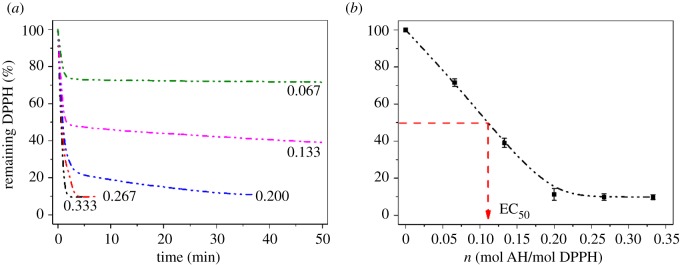
(*a*) The kinetic curves of antioxidant L^1^H_2_ with different concentrations *n*. (*b*) The curve of percentage of remaining DPPH · against concentration *n*.

The EC_50_ value of antioxidant L^1-3^H_2_ and their Fe^III^ complexes were obtained from the curves of the percentage of remaining DPPH at the steady state against the concentration *n*, where *n* is the molar ratio of antioxidant to DPPH (mol AH/mol DPPH). The percentage of remaining DPPH was determined from the kinetic curves of different concentrations *n*, figure 9 and electronic supplementary material, figures S15–S19. The EC_50_ and AE values of L^1-3^H_2_, complexes Fe^III^–L^1-3^H_2_, (BHA) butylated hydroxyanisole, catechol and ascorbic acid [46,49] are listed in table 3 (*^a^*Antioxidant. *^b^*BHA and ascorbic acid in Ref. [46]. *^c^*Catechol in Ref. [49]).

The structures of phenolic derivatives have great influence on antioxidant activity [50,51]. L^1-3^H_2_, BHA and catechol possess similar bisphenolic structures, which lead them with approximate EC_50_ values.

For the results of L^1-3^H_2_ and Fe^III^–L^1-3^H_2_, the increase in AE values can be explained by mechanism of the DPPH's reaction with phenols (ArOH). The antioxidant capacity of antioxidant L^1-3^H_2_ was influenced by amide group and coordination reaction. DPPH reacted with ArOH through an electron-transfer process from ArOH or its phenoxide anion (ArO^−^) to DPPH (electron transfer mechanism) [52]. The electron-transfer process from ArO^−^ to DPPH was fast. The presence of amide group in L^1-3^H_2_ increased the quantity of ArO^−^ and the observed values of the reaction rate, which conferred high AE values. The AE values of the complexes Fe^III^–L^1-3^H_2_ reduce generally, the Fe^III^ coordination reaction produced acids and ArO–M complex. The acids and coordination reaction all reduced the quantity of ArO^−^ and antioxidant capacity. The results were as expected.

## 3. Conclusion

In this article, a series of mono(catecholamine) derivatives, 2,3-bis(hydroxy)-*N*-(hydroxyacyl)benzamide, containing a pendant hydroxy, were synthesized and fully characterized. The results of thermodynamic stability with a set of metal ions showed that the chelators possess with high thermodynamic stability and exhibited improved selectivity to the Fe^III^ ion. Meanwhile, the results of DPPH assays of mono(catecholamine) derivatives indicated they all possess excellent antioxidant properties. These results also indicated that mono(catecholamine) derivatives have a potential application prospect as chelator for the iron overload situations without depletion of essential metal ions such as Mg^II^ and Zn^II^ ions.

## 4. Experimental section

### 4.1. General

The organic reagents used were pure commercial products from Aladdin. The solvents were purchased from Chengdu Kelong Chemical Reagents Co. The 300–400 mesh silica gels were purchased from Qingdao Hailang Chemical Reagents Co. ^1^H NMR and ^13^C NMR spectra were recorded on a Bruker Avance III 600 MHz, CDCl_3_ and CD_3_OD were used as the solvents, tetramathylsilane as the internal standard. The FTIR spectra were obtained from a Nicolet 380 FTIR spectrophotometer (Thermo Fisher Nicolet, USA) with a resolution of 4 cm^−1^ from 400 cm^−1^ to 4000 cm^−1^. The ultraviolet (UV)–vis spectrophotometer (Thermo Scientific Evolution 201, USA) used had a double-beam light source from 190to 1100 nm. Mass spectral analysis was conducted using a Varian 1200 LC/MS.

### 4.2. Synthesis of the chelators

*Synthesis of 2,3-bis(benzyloxy)benzoic acid* (**2**). A solution of 2,3-dihydroxybenzoic acid (10.20 g, 65.9 mmol), benzyl bromide (22.2 g, 130.0 mmol) and K_2_CO_3_ (18.0 g, 130.0 mmol) in acetone (220 ml) was refluxed and stirred for 24 h. After filtration, the solution was concentrated *in vacuo* to obtain the crude product as clear oil. The crude product was dissolved in methanol (200 ml), and LiOH · H_2_O (360.0 mmol, 15.1 g) was slowly added. The mixture was refluxed and stirred for 3 h. Then, the solution was acidified with 3.0 M HCl to pH 2.0 and filtered to obtain the product **2** as white solid (yield of 80%). ^1^H NMR (600 MHz, CDCl_3_): *δ* (ppm) = 7.50–7.10 (m, 12H, Ar–H), 7.03 (t, *J *= 8.0 Hz, 1H, Ar–H), 5.12 (s, 2H, O–CH2–Ar), 5.09 (s, 2H, O–CH2–Ar). ^13^C NMR (150 MHz, CDCl_3_): *δ* (ppm) = 165.38 (C = O), 151.54 (ArC), 147.32 (ArC), 136.07 (ArCH), 134.87 (ArCH), 129.51 (ArCH), 129.06 (ArCH), 129.03 (ArCH), 128.77 (ArCH), 128.00 (ArCH), 125.25 (ArCH), 124.67 (ArCH), 123.27 (ArCH), 119.21 (ArCH), 71.77 (CH_2_). FTIR (KBr, cm^−1^): 3100, 2700, 1683, 1035. APCI-MS (*m/z*): 333.4 [M-H]^-^.

*Synthesis of 2,3-bis(benzyloxy)-N-(hydroxyethyl)benzamide* (**4a**). A solution of 2,3-bis(benzyloxy)benzoic acid **2** (1.67 g, 5.0 mmol), HOBt (0.12 g, 0.9 mmol) and DCC (1.24 g, 6.0 mmol) in CH_2_Cl_2_ (50 ml) was stirred for 30 min at room temperature. Ethanolamine (0.34 g, 5.5 mmol) was added dropwise over 3 min and the mixture stirred for 10 h. The solution was filtered to remove the dicyclohexyl urea. The filtrate was concentrated *in vacuo* and the residue purified by flash column chromatography (volume ratio of acetone/hexane 2* *:* *3) to give the product **4a** as clear oil (80%). *R_f_ *= 0.4. ^1^H NMR (600 MHz, CDCl_3_): *δ* (ppm) = 8.30 (br s, 1H, CO–NH), 7.71 (m, 1H, Ar–H), 7.50-7.10 (m, 12H, Ar–H), 5.15 (s, 2H, O–CH_2_–Ar), 5.10 (s, 2H, O–CH_2_–Ar), 3.62 (t, *J* = 5.4 Hz, 2H, CH_2_), 3.40 (m, 2H, CH_2_), 2.87 (br s, 1H, OH). ^13^C NMR (150 MHz, CDCl_3_): *δ* (ppm) = 165.78 (C=O), 150.95 (ArC), 146.15 (ArC), 135.60 (ArC), 127.99 (ArCH), 127.94 (ArCH), 127.52 (ArCH), 126.92 (ArCH), 123.68 (ArCH), 122.49 (ArCH), 116.50 (ArCH), 75.72 (CH_2_), 70.56 (CH_2_), 61.88 (CH_2_), 42.10 (CH_2_). FTIR (KBr, cm^−1^): 3347, 1625, 1577, 1555, 1498, 1028. APCI-MS (*m/z*): 378.0 [M + H]^+^.

*Synthesis of 2,3-bis(benzyloxy)-N-(3-hydroxypropyl)benzamide* (**4b**). A solution of 2,3-bis(benzyloxy)benzoic acid **2** (1.67 g, 5.0 mmol), HOBt (0.12 g, 0.9 mmol) and DCC (1.24 g, 6.0 mmol) in CH_2_Cl_2_ (50 ml) was stirred for 30 min at room temperature. 3-Amino-1-propanol (0.41 g, 5.5 mmol) was added dropwise over 3 min and the mixture stirred for 10 h. The solution was filtered to remove the dicyclohexyl urea. The filtrate was concentrated *in vacuo* and the residue purified by flash column chromatography (volume ratio of acetone/hexane 2 : 3) to give the product **4b** as clear oil (85%). *R_f_* = 0.5 (volume ratio 2 : 3 acetone/hexane). ^1^H NMR (600 MHz, CDCl_3_): *δ* (ppm) = 8.12 (br s, 1H, CO–NH), 7.72 (m, 1H, Ar–H), 7.50-7.12 (m, 12H, Ar–H), 5.16 (s, 2H, O–CH_2_–Ar), 5.09 (s, 2H, O–CH_2_–Ar), 3.50 (t, *J* = 5.4 Hz, 2H, CH_2_), 3.39 (m, 2H, CH_2_), 1.52 (m, 2H, CH_2_). ^13^C NMR (150 MHz, CDCl_3_): *δ* (ppm) = 165.69 (C=O), 150.94 (ArC), 146.11 (ArC), 135.58 (ArC), 128.08 (ArCH), 127.98 (ArCH), 127.54 (ArCH), 126.89 (ArCH), 123.71 (ArCH), 122.54 (ArCH), 116.43 (ArCH), 75.76 (CH_2_), 70.55 (CH_2_), 57.89 (CH_2_), 34.89 (CH_2_), 31.69 (CH_2_). FTIR (KBr, cm^−1^): 3327, 1635, 1577, 1540, 1452, 1028. APCI-MS (*m/z*): 392.0 [M + H]^+^.

*Synthesis of 2,3-bis(benzyloxy)-N-(4-hydroxybutyl)benzamide* (**4c**). A solution of 2,3-bis(benzyloxy) benzoic acid **2** (1.67 g, 5.0 mmol), HOBt (0.12 g, 0.9 mmol) and DCC (1.24 g, 6.0 mmol) in CH_2_Cl_2_ (50 ml) was stirred for 30 min at room temperature. 4-Amino-1-butanol (0.49 g, 5.5 mmol) was added dropwise over 3 min and the mixture stirred for 10 h. The solution was filtered to remove the dicyclohexyl urea. The filtrate was concentrated *in vacuo* and the residue purified by flash column chromatography (volume ratio of acetone/hexane 2 : 3) to give the product **4c** as clear oil (90%). *R_f_* = 0.5. ^1^H NMR (600 MHz, CDCl_3_): *δ* (ppm) = 8.01 (br s, 1H, CO–NH), 7.74 (m, 1H, Ar–H), 7.50–7.10 (m, 12H, Ar–H), 5.16 (s, 2H, O–CH_2_–Ar), 5.09 (s, 2H, O–CH_2_–Ar), 3.58 (m, 2H, CH_2_), 3.32 (m, 2H, CH_2_), 1.46 (m, 4H, CH_2_–CH_2_). ^13^C NMR (150 MHz, CDCl_3_): *δ* (ppm) = 164.47.

(C=O), 150.93 (ArC), 145.99 (ArC), 135.64 (ArC), 127.99 (ArCH), 127.50 (ArCH), 126.90 (ArCH), 123.68 (ArCH), 122.52 (ArCH), 116.17 (ArCH), 75.59 (CH_2_), 70.52 (CH_2_), 61.56 (CH_2_), 38.57 (CH_2_), 29.06 (CH_2_), 25.03 (CH_2_). FTIR (KBr, cm^−1^): 3327, 1635, 1577, 1540, 1452, 1033. APCI-MS (*m/z*): 406.2 [M + H]^+^.

*Synthesis of 2,3-dihydroxy-N-(2-hydroxyethyl)benzamide* (**5a**). A mixture of **4a** (0.40 g, 1.06 mmol) and Pd/C (5%) (200 mg) in ethanol (50 ml) was stirred under H_2_ atmosphere (130 ml min^−1^) for 5 h. The resulting mixture was filtered over Celite®, evaporated to dryness and dried under vacuum to give **5a** as grey power (yield of 99%). ^1^H NMR (600 MHz, (CD_3_)_2_CO): *δ* (ppm) = 8.14 (br s, 1H, CO–NH), 7.28 (d, *J* = 7.8 Hz, 1H, Ar–H), 6.96 (dd, *J* = 7.8, 1.2 Hz,, Ar–H), 6.71 (t, *J* = 7.8 Hz, 1H, Ar–H), 3.73 (t, *J* = 6.0 Hz, 2H, CH_2_), 3.53 (m, 2H, CH_2_). ^13^C NMR (150 MHz, (CD_3_)_2_CO): *δ* (ppm) = 171.36 (C = O), 150.56 (Ar–C), 147.14 (Ar–C), 119.18 (Ar–CH), 119.03 (Ar–CH), 117.70 (Ar–CH), 115.49 (Ar–CH), 61.06 (CH_2_), 42.89 (CH_2_). FTIR (KBr, cm^−1^): 3426, 3371, 2930, 1647, 1593, 1540, 1466, 736. APCI-MS (*m/z*): 198.1 [M + H]^+^.

*Synthesis of 2,3-dihydroxy-N-(3-hydroxypropyl)benzamide* (**5b**). A mixture of **4b** (0.40 g, 1.02 mmol) and Pd/C (5%) (200 mg) in ethanol (50 ml) was stirred under H_2_ atmosphere (130 ml min^−1^) for 5 h. The resulting mixture was filtered over Celite®, evaporated to dryness and dried under vacuum to give **5b** as grey power (yield of 99%). ^1^H NMR (600 MHz, (CD_3_)_2_CO): *δ* (ppm) = 8.29 (br s, 1H, CO–NH), 7.22 (d, *J* = 7.8 Hz, 1H, Ar–H), 6.96 (dd, *J* = 7.8, 1.2 Hz,, Ar–H), 6.70 (t, *J* = 7.8 Hz, 1H, Ar–H), 3.67 (t, *J* = 6.0 Hz, 2H, CH_2_), 3.53 (m, 2H, CH_2_), 1.82 (m, 2H, CH_2_). ^13^C NMR (150 MHz, (CD_3_)_2_CO): *δ* (ppm) = 171.19 (C=O), 150.58 (Ar–C), 147.15 (Ar–C), 119.17 (Ar–CH), 119.00 (Ar–CH), 117.52 (Ar–CH), 115.49 (Ar–CH), 60.22 (CH_2_), 37.67 (CH_2_), 32.69 (CH_2_). FTIR (KBr, cm^−1^): 3366, 2933, 1639, 1589, 1548, 1460, 741. APCI-MS (*m/z*): 212.2 [M + H]^+^.

*Synthesis of 2,3-dihydroxy-N-(3-hydroxybutyl)benzamide* (**5c**). A mixture of **4c** (0.40 g, 0.99 mmol) and Pd/C (5%) (200 mg) in ethanol (50 ml) was stirred under H_2_ atmosphere (130 ml min^−1^) for 5 h. The resulting mixture was filtered over Celite®, evaporated to dryness and dried under vacuum to give **5c** as grey power (yield of 99%). ^1^H NMR (600 MHz, (CD_3_)_2_CO): *δ* (ppm) = 8.29 (br s, 1H, CO–NH), 7.26 (d, *J* = 7.8 Hz, 1H, Ar–H), 6.96 (dd, *J* = 7.8, 1.2 Hz, Ar–H), 6.69 (t, *J* = 7.8 Hz, 1H, Ar–H), 3.60 (t, *J* = 6.0 Hz, 2H, CH_2_), 3.43 (m, 2H, CH_2_), 1.70 (m, 2H, CH_2_), 1.60 (m, 2H, CH_2_). ^13^C NMR (150 MHz, (CD_3_)_2_CO): *δ* (ppm) = 171.05 (C=O), 150.57 (Ar–C), 147.08 (Ar–C), 119.10 (Ar–CH), 118.92 (Ar–CH), 117.55 (Ar–CH), 115.55 (Ar–CH), 62.02 (CH_2_), 39.98 (CH_2_), 30.27 (CH_2_), 26.60 (CH_2_). FTIR (KBr, cm^−1^): 3409, 3238, 2954, 1644, 1583, 1543, 1475, 763. APCI-MS (*m/z*): 226.1 [M + H]^+^.

### 4.3. Titration solution and methods

An INESA ZDJ-4B automatic potential titrator was used to measure the pH of the experimental solutions. Meanwhile, it was used for incremental additions of base standard solution to the titration cup under N_2_ atmosphere. Titrations were performed in 0.10 M KCl supporting electrolyte. The temperature of the experimental solution was maintained at 298.2 K by an externally thermostat water bath. UV–visible spectra for incremental titrations and batch titrations were recorded on a Thermo Scientific Evolution 201 UV–vis spectrophotometer. Solid reagents were weighed on a Sartorius BT25S analytical balance accurate to 0.01 mg. All titration solutions were prepared using distilled water from Ulupure ULUP-IV ultra water system and degassed by an ultrasonic device. Standard solution of 0.10 M KOH and HNO_3_ were purchased from Aladdin. Chelator stock solutions were made by dissolving a weighed amount of chelator accurate to 0.01 mg in 0.10 M KCl supporting electrolyte in a volumetric flask. A stock solution of 2.5 × 10^−3^ M metal ion (Fe^III^, Mg^II^ and Zn^II^ ions) was made by dissolving a weighed amount of corresponding metal salt (FeCl_3_, ZnCl_2_ and MgCl_2_ · 6H_2_O 99.95% metals basis) in 1.0 vol % HNO_3_ standard solution. Fe^III^ ion titrations were conducted with a 3 : 1 chelator : metal ratio. Mg^II^ and Zn^II^ ions titrations were conducted with a 1 : 1 chelator : metal ratio. Metal-to-chelator ratios were controlled by careful addition of a chelator solution of known concentration and a metal ion stock solution to the titration cup. All titrations were repeated a minimum of three times.

### 4.4. Titration and treatment

Spectrophotometric titration data were analysed using the program HypSpec 2014 [27], using nonlinear least-squares regression to determine formation constants. Wavelengths between 400–800 nm of Fe^III^ titration curves and 250–550 nm of Mg^II^, Zn^II^ titration curves were used for data refinement. The number of absorbing species to be refined upon was determined by factor analysis within the HypSpec 2014 [27]. Speciation diagrams were generated using HySS [37] titration simulation software. The protonation constants and metal complex formation constants were determined by potentiometric and spectrophotometric titration experiments.

### 4.5. Antioxidant assay methods

An aliquot of methanol (0.1 ml) and different aliquot stock methanol solutions of 1.0 × 10^−4^ M antioxidant were added to the 2.5 ml methanol solution of 6.0 × 10^−5^ M DPPH, and the volume was adjusted to a final value of 3.0 ml with methanol. Absorbances at 517 nm were measured immediately at 10 s intervals on a Thermo Scientific Evolution 201 UV–vis spectrophotometer until the reaction reached steady state. Five different concentrations were measured for each assay. Then the EC_50_ were plotted to obtain from the graph the percentage of remaining DPPH at the steady state against the molar ratio antioxidant to DPPH. Moreover, the time needed to reach the steady state to EC_50_ concentration (*T*_EC50_) and the AE values was also calculated.

## Supplementary Material

The Mono(catecholamide) Derivatives as Iron Chelators. Synthesis, Solution Thermodynamic Stability, and Antioxidant Research

## Data accessibility

This article does not contain any additional data.

## References

[1] Zecca L, Youdim MB, Riederer P, Connor JR, Crichton RR 2004 Iron, brain ageing and neurodegenerative disorders. Nat. Rev. Neurosci. 5, 863–873. (doi:10.1038/nrn1537) 15496864 10.1038/nrn1537

[2] Berg D, Gerlach M, Youdim M, Double K, Zecca L, Riederer P, Becker G 2001 Brain iron pathways and their relevance to Parkinson's disease. J. Neurochem. 79, 225–236. (doi:10.1046/j.1471-4159.2001.00608.x) 11677250 10.1046/j.1471-4159.2001.00608.x

[3] Ward RJ, Dexter DT, Crichton RR 2015 Neurodegenerative diseases and therapeutic strategies using iron chelators. J. Trace Elem. Med. Biol. 31, 267–273. (doi:10.1016/j.jtemb.2014.12.012) 25716300 10.1016/j.jtemb.2014.12.012

[4] Workman DG et al. 2015 Protection from neurodegeneration in the 6-hydroxydopamine (6-OHDA) model of Parkinson's with novel 1-hydroxypyridin-2-one metal chelators. Metallomics 7, 867–876. (doi:10.1039/C4MT00326H) 25781076 10.1039/c4mt00326h

[5] Harris WR, Carrano CJ, Cooper SR, Sofen SR, Avdeef AE, Mcardle JV, Raymond KN 1979 Coordination chemistry of microbial iron transport compounds. 19. Stability constants and electrochemical behavior of ferric enterobactin and model complexes. J. Am. Chem. Soc. 101, 6097–6104. (doi:10.1021/ja00514a037)

[6] Raymond KN, Freeman GE, Kappel MJ 1984 Actinide-specific complexing agents: their structural and solution chemistry. Inorg. Chim. Acta 94, 193–204. (doi:10.1016/S0020-1693(00)88005-6)

[7] Weitl FL, Raymond KN 1979 Ferric ion sequestering agents. 1. Hexadentate O-bonding *N,N’,N''*-tris(2,3-dihydroxybenzoyl) derivatives of 1,5,9-triazacyclotridecane and 1,3,5-triaminomet hylbenzene. J. Am. Chem. Soc. 101, 2728–2731. (doi: 10.1021/ja00504a039)

[8] Kappel MJ, Raymond KN 1982 Ferric ion sequestering agents. 10. Selectivity of sulfonated poly (catechoylamides) for ferric ion. Inorg. Chem. 21, 3437–3442. (doi:10.1021/ic00139a034)

[9] Preisig-Müller R, Schwekendiek A, Brehm I, Reif HJ, Kindl H 2015 Piperazine derivatives as iron chelators: a potential application in neurobiology. Biol. Met. 28, 1043–1061. (doi:10.1007/s10534-015-9889-x) 10.1007/s10534-015-9889-x 26502356

[10] Guerra KP, Delgado R 2008 Homo- and heterodinuclear complexes of the tris (catecholamide) derivative of a tetraazamacrocycle with Fe^3+^, Cu^2+^ and Zn^2+^ metal ions. Dalton Trans. 4, 539–550. (doi:10.1039/B712916E) 10.1039/b712916e 18185872

[11] Zhou T, Hider RC, Kong X 2015 Mode of iron(III) chelation by hexadentate hydroxypyridinones. Chem. Commun. 51, 5614–5617. (doi:10.1039/c4cc10339d) 10.1039/c4cc10339d 25611052

[12] Zhou T, Liu ZD, Neubert H, Kong XL, Ma YM, Hider RC 2005 High affinity iron(III) scavenging by a novel hexadentate 3-hydroxypyridin-4-one-based dendrimer: synthesis and characterization. Bioorg. Med. Chem. Lett. 15, 5007–5011. (doi:10.1016/j.bmcl.2005.08.008) 16153843 10.1016/j.bmcl.2005.08.008

[13] Zhou YJ, Liu MS, Osamah AR, Kong XL, Alsam S, Battah S, Xie YY, Hider RC, Zhou T 2015 Hexadentate 3-hydroxypyridin-4-ones with high iron(III) affinity: design, synthesis and inhibition on methicillin resistant *Staphylococcus aureus* and *Pseudomonas* strains. Eur. J. Med. Chem. 94, 8–21. (doi:10.1016/j.ejmech.2015.02.050) 25747496 10.1016/j.ejmech.2015.02.050

[14] Xu J, O'Sulliva B, Raymond KN 2002 Hexadentate hydroxypyridonate iron chelators based on TREN-Me-3, 2-HOPO: variation of cap size. Inorg. Chem. 41, 6731–6742. (doi:10.1021/ic025610+) 12470069 10.1021/ic025610+

[15] Piyamongkol S, Ma YM, Kong XL, Liu ZD, Aytemir MD, van der Helm D, Hider RC 2010 Amido-3-hydroxypyridin-4-ones as iron(III) ligands. Chemistry 16, 6374–6381. (doi:10.1002/chem.200902455) 20397153 10.1002/chem.200902455

[16] Propper RD, Shurin SB, Nathan DG 1976 Reassessment of the use of desferrioxamine B in iron overload. N. Engl. J. Med. 294, 1421–1423. (doi:10.1056/NEJM197606242942603) 1272274 10.1056/NEJM197606242942603

[17] Lipinski CA, Lombardo F, Dominy BW, Feeney PJ 1997 Experimental and computational approaches to estimate solubility and permeability in drug discovery and development settings. Adv. Drug Deliver. Rev. 23, 3–25. (doi:10.1016/S0169-409X(00)00129-0) 10.1016/s0169-409x(00)00129-0 11259830

[18] Martinbastida A et al. 2017 Brain iron chelation by deferiprone in a phase 2 randomised double-blinded placebo controlled clinical trial in Parkinson's disease. Sci. Rep. 7, 1398 (doi:10.1038/s41598-017-01402-2) 28469157 10.1038/s41598-017-01402-2 PMC5431100

[19] Galanello R, Campus S 2009 Deferiprone chelation therapy for thalassemia major. Acta Haematol. 122, 155–164. (doi:10.1159/000243800) 19907153 10.1159/000243800

[20] Barman-Balfour JA, Foster RH 1999 Deferiprone: a review of its clinical potential in iron overload in beta-thalassaemia major and other transfusion-dependent diseases. Drugs 58, 553–578. (doi:10.2165/00003495-199958030-00021) 10493280 10.2165/00003495-199958030-00021

[21] Tricta F, Uetrecht J, Galanello R, Connelly J, Rozova A, Spino M, Palmblad J 2016 Deferiprone-induced agranulocytosis: 20 years of clinical observations. Am. J. Hematol. 91, 1026–1031. (doi:10.1002/ajh.24479) 27415835 10.1002/ajh.24479 PMC5129477

[22] Zhou T, Ma Y, Kong X, Hider RC 2012 Design of iron chelators with therapeutic application. Dalton Trans. 41, 6371–6389. (doi:10.1039/c2dt12159j) 22391807 10.1039/c2dt12159j

[23] Dhungana S, White PS, Crumbliss AL 2001 Crystal structure of ferrioxamine B: a comparative analysis and implications for molecular recognition. JBIC J. Biol. Inorg. Chem. 6, 810–818. (doi:10.1007/s007750100259) 11713688 10.1007/s007750100259

[24] Khodr HH, Hider RC, Duhme-Klair AK 2002 The iron-binding properties of aminochelin, the mono(catecholamide) siderophore of *Azotobacter vinelandii*. JBIC J. Biol. Inorg. Chem. 7, 891–896. (doi:10.1007/s00775-002-0375-x) 12203027 10.1007/s00775-002-0375-x

[25] Laursen B, Denieul MP, Skrydstrup T 2002 Formal total synthesis of the PKC inhibitor, balanol: preparation of the fully protected benzophenone fragment. Tetrahedron 58, 2231–2238. (doi:10.1016/S0040-4020(02)00096-0)

[26] Gardner RA, Kinkade R, Wang C, Th PO 2004 Total synthesis of petrobactin and its homologues as potential growth stimuli for *Marinobacter hydrocarbonoclasticus*, an oil-degrading bacteria. J. Org. Chem. 69, 3530–3537. (doi:10.1021/jo049803l) 15132566 10.1021/jo049803l

[27] Gans P, Sabatini A, Vacca A 1999 Determination of equilibrium constants from spectrophometric data obtained from solutions of known pH: the program pHab. Ann. Chim. 89, 45–49.

[28] Gans P, Sabatini A, Vacca A 1996 Investigation of equilibria in solution. Determination of equilibrium constants with the HYPERQUAD suite of programs. Talanta 43, 1739–1753. (doi:10.1016/0039-9140(96)01958-3) 18966661 10.1016/0039-9140(96)01958-3

[29] Salama S, Stong JD, Neilands JB, Spiro TG 1978 Electronic and resonance Raman spectra of iron(III) complexes of enterobactin, catechol, and *N*-methyl-2,3-dihydroxybenzamide. Biochemistry 17, 3781–3785. (doi:10.1021/bi00611a017) 151556 10.1021/bi00611a017

[30] Charkoudian LK, Franz KJ 2006 Fe(III)-coordination properties of neuromelanin components: 5,6-dihydroxyindole and 5,6-dihydroxyindole-2-carboxylic acid. Inorg. Chem. 45, 3657–3664. (doi:10.1021/ic060014r) 16634598 10.1021/ic060014r

[31] Huang SP, Franz KJ, Olmstead MM, Fish RH 1995 Synthetic and structural studies of a linear Bis-catechol amide, *N,N'*-Bis(2,3-dihydroxybenzoyl)-1,7-diazaheptane (5-LICAM), and its complexes with Ni^2+^ and Co^2+^: utilization of a polymer-supported, sulfonated analog, 5-LICAMS, as a biomimetic ligand for divalent metal ion removal from aqueous solution. Inorg. Chem. 34, 2820–2825. (doi:10.1021/ic00115a007)

[32] Xu J, Kullgren B, Durbin PW, Raymond KN 1995 Specific sequestering agents for the actinides. 28. Synthesis and initial evaluation of multidentate 4-carbamoyl-3-hydroxy-1-methyl-2(1H)-pyridinone ligands for *in vivo* plutonium(IV) chelation. J. Med. Chem. 38, 2606–2614. (doi:10.1021/jm00014a013) 7629800 10.1021/jm00014a013

[33] Hay BP, Dixon DA, Vargas R, Garza J, Raymond KN 2001 Structural criteria for the rational design of selective ligands. 3. Quantitative structure-stability relationship for iron (III) complexation by tris-catecholamide siderophores. Inorg. Chem. 40, 3922–3935. (doi:10.1021/ic001380s) 11466050 10.1021/ic001380s

[34] Nardillo AM 1981 Equilibrium constants of the Fe(III)-catechol-dodecyltrimethylammonium ion system. J. Inorg. Nucl. Chem. 43, 620–624. (doi:10.1016/0022-1902(81)80522-2)

[35] Karpishin TB, Gebhard MS, Solomon EI, Raymond KN 1991 Spectroscopic studies of the electronic structure of iron(III) tris(catecholates). J. Am. Chem. Soc. 113, 2977–2984. (doi:10.1021/ja00008a028)

[36] Sever MJ, Wilker JJ 2004 Visible absorption spectra of metal-catecholate and metal-tironate complexes. Dalton Trans. 7, 1061–1072. (doi:10.1039/b315811j) 10.1039/b315811j 15252685

[37] Alderighi L, Gans P, Ienco A, Peters D, Sabatini A, Vacca A 1999 Hyperquad simulation and speciation (HySS): a utility program for the investigation of equilibria involving soluble and partially soluble species. Coord. Chem. Rev. 184, 311–318. (doi:10.1016/S0010-8545(98)00260-4)

[38] Nurchi VM, Crisponi G, Pivetta T, Donatoni M, Remelli M 2008 Potentiometric, spectrophotometric and calorimetric study on iron(III) and copper(II) complexes with 1,2-dimethyl-3-hydroxy-4-pyridinone. J. Inorg. Biochem. 102, 684–692. (doi:10.1016/j.jinorgbio.2007.10.012) 18061272 10.1016/j.jinorgbio.2007.10.012

[39] Santos MA, Gama S, Gano L, Cantinho G, Farkas E 2004 A new bis(3-hydroxy-4-pyridinone)-IDA derivative as a potential therapeutic chelating agent. Synthesis, metal-complexation and biological assays. Dalton Trans. 21, 3772–3781. (doi:10.1039/B409357G) 10.1039/B409357G 15510305

[40] Zhang QC et al. 2017 New tris(dopamine) derivative as an iron chelator. Synthesis, solution thermodynamic stability, and antioxidant research. J. Inorg. Biochem. 171, 29–36. (doi:10.1016/j.jinorgbio.2017.03.003) 28364616 10.1016/j.jinorgbio.2017.03.003

[41] Biaso F, Baret P, Pierre JL, Serratrice G 2002 Comparative studies on the iron chelators O-TRENSOX and TRENCAMS: selectivity of the complexation towards other biologically relevant metal ions and Al^3+^. J. Inorg. Biochem. 89, 123–130. (doi:10.1016/S0162-0134(01)00401-9) 11931972 10.1016/s0162-0134(01)00401-9

[42] Athavale VT, Prabhu LH, Vartak DG 1966 Solution stability constants of some metal complexes of derivatives of catechol. J. Inorg. Nucl. Chem. 28, 1237–1249. (doi:10.1016/0022-1902(66)80450-5)

[43] Zhang QC, Jin B, Shi ZT, Wang XF, Liu QQ, Lei S, Peng RF 2016 Novel enterobactin analogues as potential therapeutic chelating agents: synthesis, thermodynamic and antioxidant studies. Sci. Rep. 6, 34024 (doi:10.1038/srep34024) 27671769 10.1038/srep34024 PMC5037427

[44] Lei S, Jin B, Zhang QC, Zhang ZC, Wang XF, Peng RF, Chu SJ 2016 Synthesis of bifunctional biscatecholamine chelators for uranium decorporation. Polyhedron 119, 387–395. (doi:10.1016/j.poly.2016.09.006)

[45] Blois MS 1958 Antioxidant determination by use of stable free radicals. Nature 181, 1199–1200. (doi:10.1038/1811199a0)

[46] Sánchez-Moreno C, Larrauri JA, Saura-Calixto F 1998 A procedure to measure the antiradical efficiency of polyphenols. J. Sci. Food Agric. 76, 270–276. (doi:10.1002/(SICI)1097-0010(199802)76:2<270::AID-JSFA945>3.0.CO;2-9)

[47] Sharma OP, Bhat TK 2009 DPPH antioxidant assay revisited. Food Chem. 113, 1202–1205. (doi:10.1016/j.foodchem.2008.08.008)

[48] Romano CS, Abadi K, Repetto V, Vojnov AA, Moreno S 2009 Synergistic antioxidant and antibacterial activity of rosemary plus butylated derivatives. Food Chem. 115, 456–461. (doi:10.1016/j.foodchem.2008.12.029)

[49] Bortolomeazzi R, Sebastianutto N, Toniolo R, Pizzariello A 2007 Comparative evaluation of the antioxidant capacity of smoke flavouring phenols by crocin bleaching inhibition, DPPH radical scavenging and oxidation potential. Food Chem. 100, 1481–1489. (doi:10.1016/j.foodchem.2005.11.039)

[50] Cuvelier ME, Richard H, Berset C 1992 Comparison of the antioxidative activity of some acid-phenols: structure-activity relationship. Biosci. Biotechnol. Biochem. 56, 324–325. (doi:10.1271/bbb.56.324)

[51] Shahidi F, Wanasundara PK 1992 Phenolic antioxidants. Crit. Rev. Food Sci. Nutr. 32, 67–103. (doi:10.1016/S0924-2244(00)89114-1) 1290586 10.1080/10408399209527581

[52] Foti MC, Daquino C, Geraci C 2004 Electron-transfer reaction of cinnamic acids and their methyl esters with the DPPH radical in alcoholic solutions. J. Org. Chem. 69, 2309–2314. (doi:10.1021/jo035758q) 15049623 10.1021/jo035758q

