# Supplementary material for: The mono(catecholamine) derivatives as iron chelators: synthesis, solution thermodynamic stability and antioxidant properties research
Source: R Soc Open Sci. 2018 Jun 6;5(6):171492. doi: 10.1098/rsos.171492 (PMC6030290; doi:10.1098/rsos.171492)
Supplement: The Mono(catecholamide) Derivatives as Iron Chelators. Synthesis, Solution Thermodynamic Stability, and Antioxidant Research [file rsos171492supp1.docx]

The Mono(catecholamine) Derivatives as Iron Chelators. Synthesis, Solution Thermodynamic Stability, and Antioxidant Research

Qingchun Zhang^1,2^, Bo Jin^1,^*, Xiaofang Wang^1^, Shan Lei^1^, Zhaotao Shi^2^, Jia Zhao^2^, Qiangqiang Liu^3^, Rufang Peng^1,^*

*^1^ State Key Laboratory Cultivation Base for Nonmetal Composites and Functional Materials, Southwest University of Science and Technology, Mianyang 621010, China;*

*^2^ School of Materials Science and Engineering, Southwest University of Science and Technology, Mianyang 621010, China;*

*^3^ Research Center of Laser Fusion, China Academy of Engineering Physics, Mianyang 621010, China;*

******* *Correspondence:* [*jinbo0428@163.com*](mailto:jinbo0428@163.com) *(Bo Jin) and* [*pengrufang@swust.edu.cn (R*](mailto:pengrufang@swust.edu.cn%20(R)*ufang Peng); Tel.: 86-816-2419011*





**Figure S1.** The spectrophotometric titration spectrogram of L^2^H_2_, condition: [L^2^H_2_] = 5.0 × 10^-4^ M, *µ* = 0.10 M KCl, *T* = 298.2 K, pH range = 5.4-12.4.





**Figure S2.** The spectrophotometric titration spectrogram of L^3^H_2_, condition: [L^3^H_2_] = 5.0 × 10^-4^ M, *µ* = 0.10 M KCl, *T* = 298.2 K, pH range = 5.4-12.5.





**Figure S3.** Species distribution curves of chelator L^2^H_2_, calculative condition: [L^2^H_2_] = 5.0 × 10^-4^ M, the charge numbers are omitted for clarity.





**Figure S4.** Species distribution curves of chelator L^3^H_2_, calculative condition: [L^3^H_2_] = 5.0 × 10^-4^ M, the charge numbers are omitted for clarity.





**Figure S5.** The spectrophotometric titration spectrogram of Fe^3+^-L^2^H_2_ complex, condition: [L^2^H_2_] = 3 × [Fe^3+^] = 2.0 × 10^-4^ M, *µ* = 0.10 M KCl, *T* = 298.2 K, pH range = 3.86-11.02.





**Figure S6.** The spectrophotometric titration spectrogram of Fe^3+^-L^3^H_2_ complex, condition: [L^3^H_2_] = 3 × [Fe^3+^] = 2.0 × 10^-4^ M, *µ* = 0.10 M KCl, *T* = 298.2 K, pH range = 3.88-11.06.





**Figure S7.** Species distribution curves of Fe^3+^-L^2^H_2_ complex, calculative condition: [L^2^H_2_] = 3 × [Fe^3+^] = 2.0 × 10^-4^ M, the charge numbers are omitted for clarity.





**Figure S8.** Species distribution curves of Fe^3+^-L^3^H_2_ complex, calculative condition: [L^3^H_2_] = 3 × [Fe^3+^] = 2.0 × 10^-4^ M, the charge numbers are omitted for clarity.





**Figure S9.** The spectrophotometric titration spectrogram of Mg^2+^-L^1^H_2_ complex, condition: [L^1^H_2_] = [Mg^2+^] = 2.0 × 10^-4^ M, *µ* = 0.10 M KCl, *T* = 298.2 K, pH range = 5.65-11.23.





**Figure S10.** The spectrophotometric titration spectrogram of Mg^2+^-L^2^H_2_ complex, condition: [L^2^H_2_] = [Mg^2+^] = 2.0 × 10^-4^ M, *µ* = 0.10 M KCl, *T* = 298.2 K, pH range = 5.71-11.32.





**Figure S11.** The spectrophotometric titration spectrogram of Mg^2+^-L^3^H_2_ complex, condition: [L^3^H_2_] = [Mg^2+^] = 2.0 × 10^-4^ M, *µ* = 0.10 M KCl, *T* = 298.2 K, pH range = 5.84-11.26.





**Figure S12.** The spectrophotometric titration spectrogram of Zn^2+^-L^1^H_2_ complex, condition: [L^1^H_2_] = [Zn^2+^] = 2.0 × 10^-4^ M, *µ* = 0.10 M KCl, *T* = 298.2 K, pH range = 5.64-11.10.





**Figure S13.** The spectrophotometric titration spectrogram of Zn^2+^-L^2^H_2_ complex, condition: [L^2^H_2_] = [Zn^2+^] = 2.0 × 10^-4^ M, *µ* = 0.10 M KCl, *T* = 298.2 K, pH range = 5.55-11.11.





**Figure S14.** The spectrophotometric titration spectrogram of Zn^2+^-L^3^H_2_ complex, condition: [L^3^H_2_] = [Zn^2+^] = 2.0 × 10^-4^ M, *µ* = 0.10 M KCl, *T* = 298.2 K, pH range = 6.00-11.13.


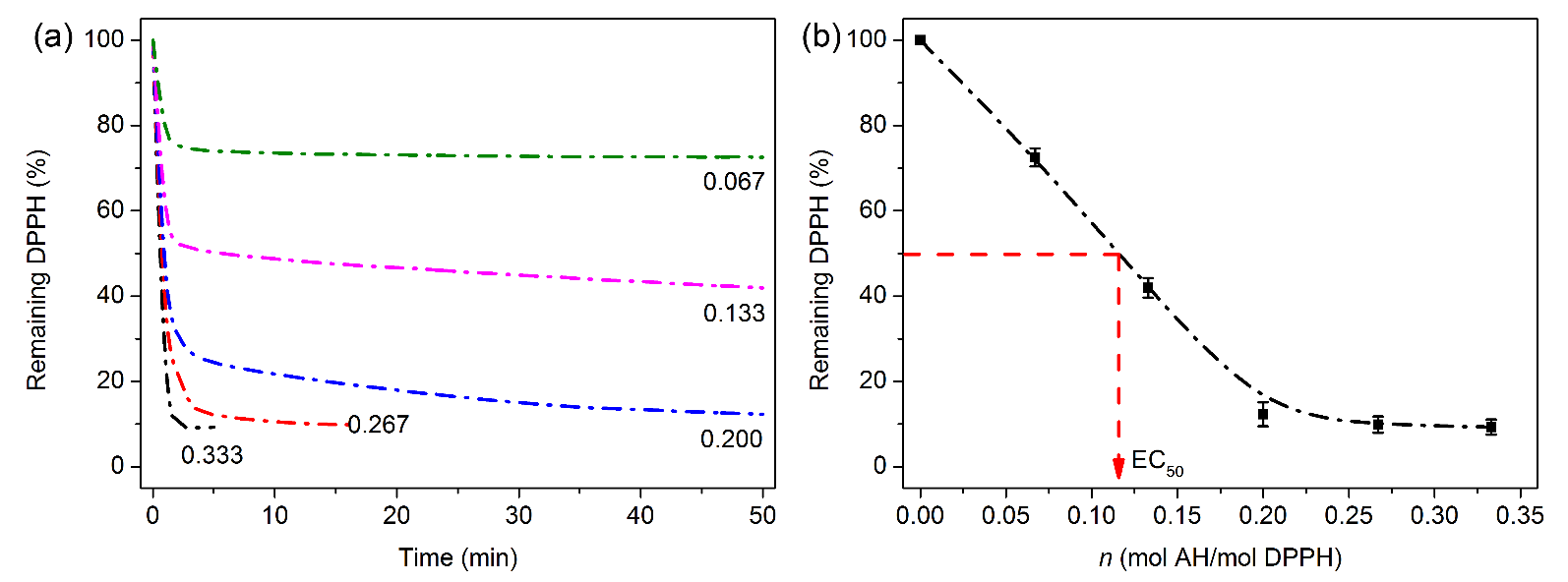


**Figure S15.** (a) The kinetic curves of antioxidant L^2^H_2_ with different concentrations *n*, (b) The curve of percentage of remaining DPPH·against concentration *n*.


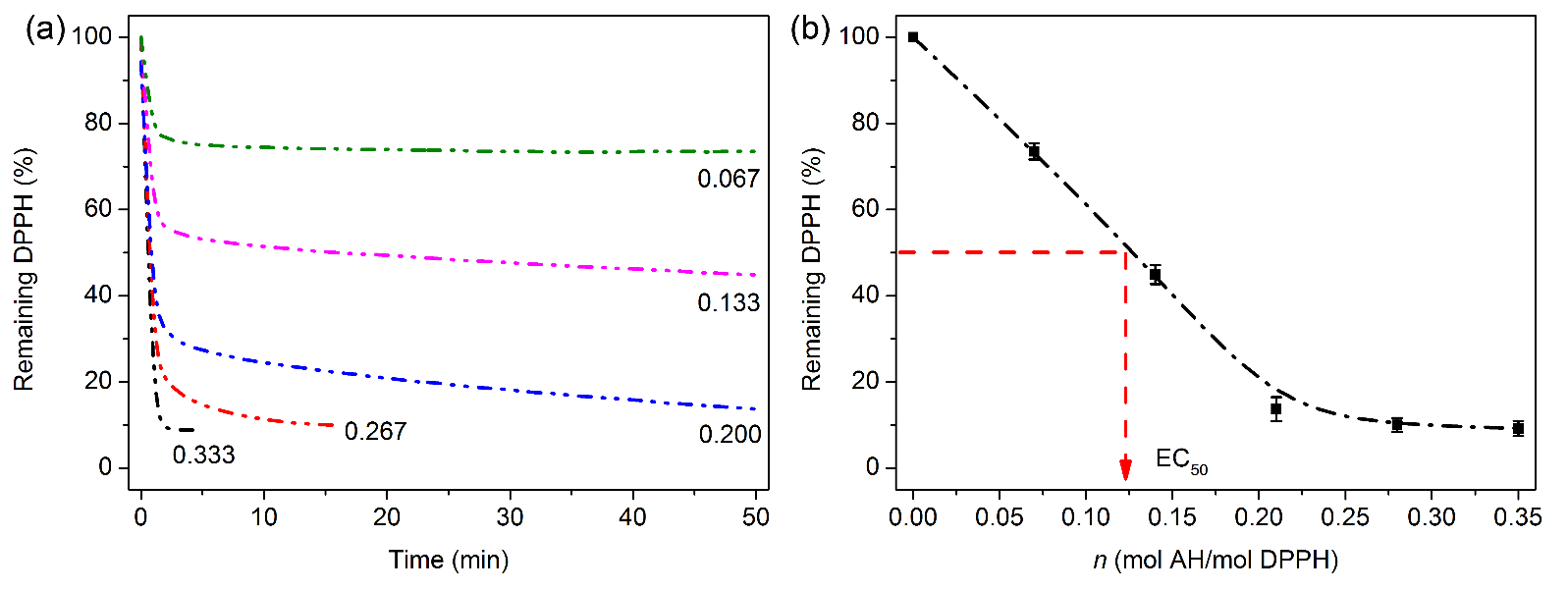


**Figure S16.** (a) The kinetic curves of antioxidant L^3^H_2_ with different concentrations *n*, (b) The curve of percentage of remaining DPPH·against concentration *n*.


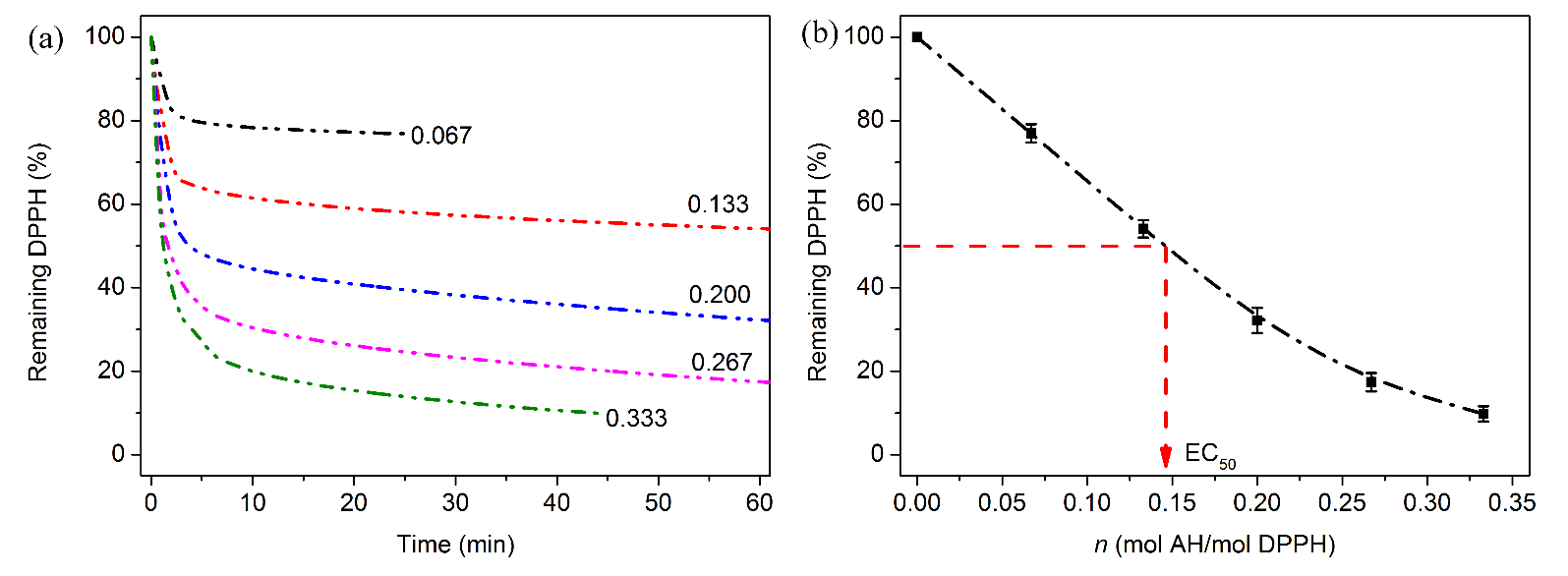


**Figure S17.** (a) The kinetic curves of complex Fe^III^-L^1^H_2_ with different concentrations *n*, (b) The curve of percentage of remaining DPPH·against concentration *n*.


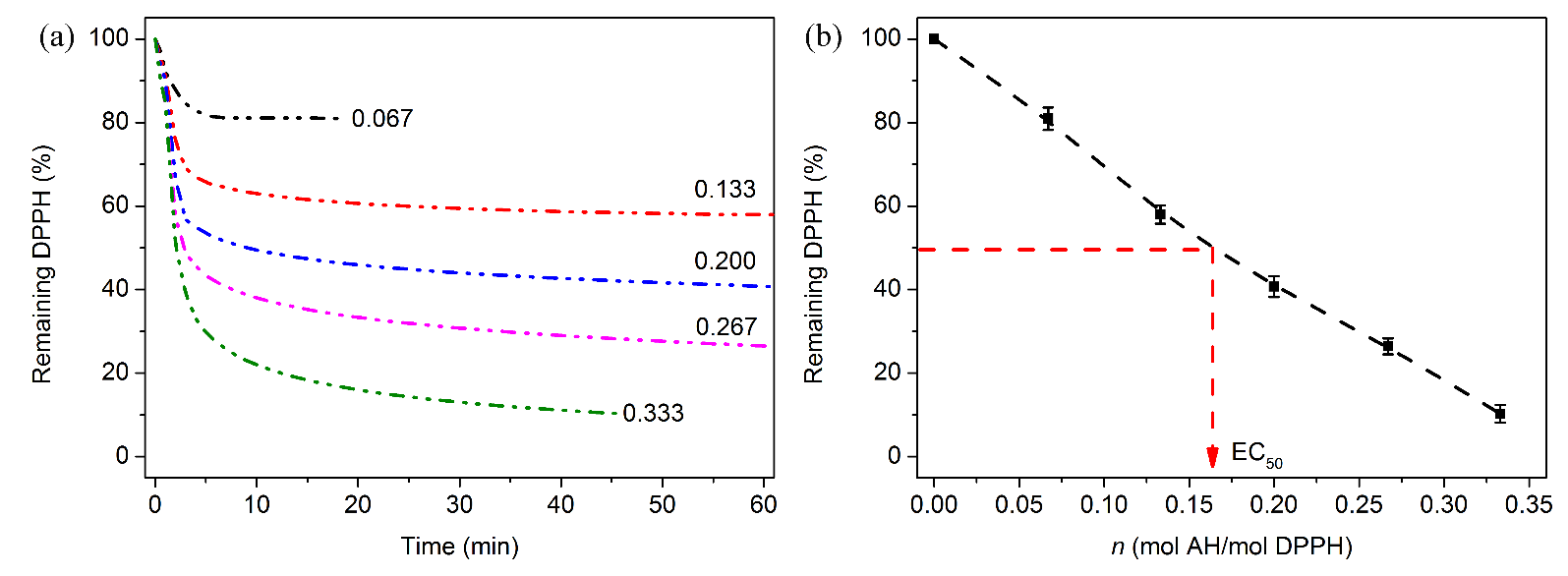


**Figure S18.** (a) The kinetic curves of complex Fe^III^-L^2^H_2_ with different concentrations *n*, (b) The curve of percentage of remaining DPPH·against concentration *n*.


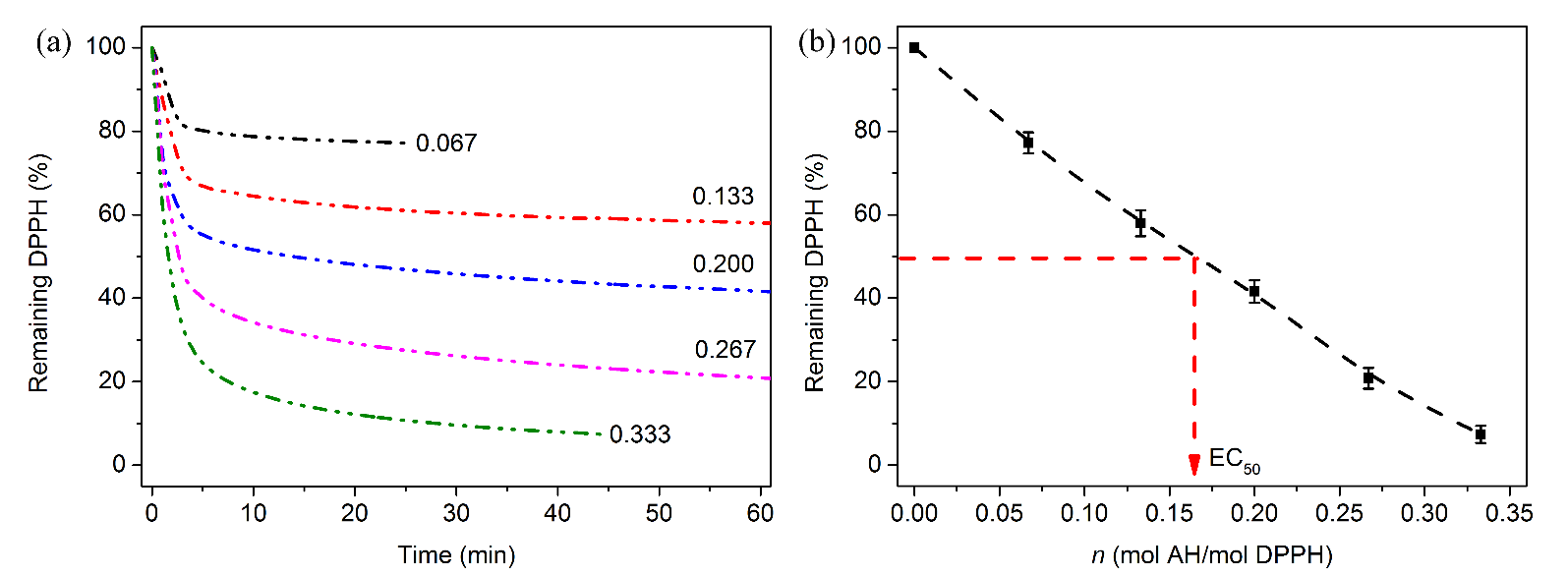


**Figure S19.** (a) The kinetic curves of complex Fe^III^-L^3^H_2_ with different concentrations *n*, (b) The curve of percentage of remaining DPPH·against concentration *n*.
